# Chronic cluster‐like headache in a patient with a macroprolactinoma presenting with falsely low prolactin levels: bromocriptine versus cabergoline?

**DOI:** 10.1002/ccr3.1208

**Published:** 2017-09-29

**Authors:** Maria M. Pineyro, Gabriela Sosa, Maria R. Finozzi, Natalia Stecker, Raul Pisabarro, Maria C. Belzarena

**Affiliations:** ^1^ Clínica de Endocrinología y Metabolismo Hospital de Clínicas Facultad de Medicina Universidad de la República Montevideo Uruguay

**Keywords:** Bromocriptine, cabergoline, cluster‐like headache, hook effect, macroprolactinoma

## Abstract

Cluster‐like headache may be associated with pituitary tumors, mostly prolactinomas. Pituitary imaging and prolactin measurement should be assessed in patients presenting with cluster‐like headaches with atypical features or unsatisfactory response to treatment. Furthermore, large pituitary adenomas with moderate increase in prolactin levels should prompt prolactin dilutions to avoid “hook effect”.

## Introduction

Cluster headache is characterized by recurrent attacks of severe or very severe pain, with unilateral localization in orbital, supraorbital, and/or temporal sites, lasting 15–180 min if untreated. It is accompanied by at least one ipsilateral symptom of autonomic dysfunction such as nasal congestion and/or rhinorrhoea, conjunctival injection and/or lacrimation, forehead and facial sweating, eyelid edema, miosis and/or ptosis, and a sense of restlessness or agitation. In the episodic form, attacks are clustered in periods of weeks and months with remission periods of months or years. In the chronic form, attacks happen for more than 1 year without remission or with periods of remission that last <1 month 1. The trigeminal system is presumed to be involved.

The diagnosis of primary cluster headache involves ruling out disorders of nervous system. Cluster‐like headaches have been reported with a variety of structural neurological alterations. It has been reported that it was associated with pituitary adenomas, mostly with prolactinomas.

We report a case of macroprolactinoma presenting with cluster‐like headaches, with increased frequency associated with use of cabergoline, but relieved with bromocriptine. Furthermore, this macroprolactinoma presented initially with moderate hyperprolactinemia due to “hook effect”.

## Case History

An 18‐year‐old Caucasian female without a significant past medical history presented with complaints of recurrent headaches for the last 3 years. The headaches were described as sharp left retroorbital pain, intense, associated with ipsilateral lacrimation, conjunctival injection, and ptosis. The attacks lasted around 2 h, occasionally for longer periods of time. The headaches occurred 4–5 times/week, with no known triggering factors and no remission periods. She was evaluated by a neurologist and the initial neurological examination was normal. She was treated with nonsteroidal anti‐inflammatory drugs and ergotamine, decreasing frequency of attacks to 1–2 times/week.

One year later, she presented with secondary amenorrhea. Evaluated by gynecologist she was started on OCP, which she received for 3 months. Later on she noticed bilateral galactorrhea. She consulted endocrinologist, who measured prolactin levels (33.1 ng/mL, reference range 3–23), and was referred to our department.

Physical examination showed positive bilateral galactorrhea with expression. Neurological examination was still normal, with normal visual field testing on confrontation.

**Figure 1:**
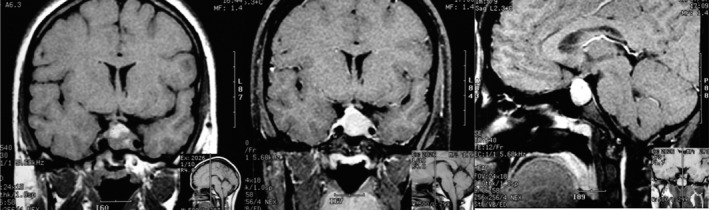
Coronal and sagittal T1 weighted pituitary MRI image at diagnosis shows a sellar mass with optic chiasmal compression and left cavernous sinus invasion.

**Figure 2:**
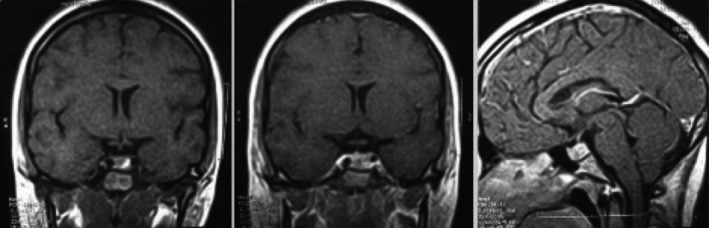
Coronal and sagittal T1 weighted MRI image 3 months after bromocriptine treatment shows a decrease in tumor size.

Initial laboratory tests showed prolactin levels measured by chemiluminescence's assay were 125 ng/mL (*N* 2.8–29.2). At diagnosis, TSH was 2.11 mUI/mL (*N* 0.27–4.20), FT4 1.02 ng/dL (*N* 0.93–1.70), and morning cortisol 17.43 *μ*g/dL (*N* > 15). Age‐adjusted IGF‐1 was normal. Formal Godlmann visual field perimetry showed upper bitemporal quadrantanopia. Brain magnetic resonance image (MRI) revealed a lobulated sellar mass measuring 30 × 20 mm and homogeneously enhancing after gadolinium, with suprasellar, sphenoid, and left cavernous sinus invasion and optic chiasm compression (Fig. 1). Repeated prolactin level performing a 1/100 dilution was 1320 ng/mL. The patient was diagnosed with a macroprolactinoma with left cavernous sinus invasion, presenting with cluster‐like headaches and moderate hyperprolactinemia due to “hook effect”. Patient was started on cabergoline with gradual increase to 1 mg per week, which elicited severe headaches attacks with similar characteristics to previous ones, but lasting 24 h. After 1 month, she was switched to low doses of bromocriptine, with gradual increase till 7.5 mg/day. Bromocriptine was well tolerated, becoming asymptomatic immediately after initiating treatment. Two months later, menstruation cycles returned. After 1 month of bromocriptine, prolactin levels normalized at 11.5 ng/mL and remained normal 2 years later. Three months after starting bromocriptine new MRI showed decrease size of pituitary tumor, measuring 12 × 12 mm with slight deformation of its superior border with heterogeneous intensity in T1 and T2; there was no clear delimitation of left cavernous sinus. There was a slight deviation of stalk to the right. There were no suprasellar extension or optic chiasm alterations (Fig. 2). One and half years later, new MRI showed decrease size of pituitary tumor, measuring 5 × 3 mm, without cavernous sinus involvement.

## Discussion

This case is remarkable for a macroprolactinoma presenting with cluster‐like headaches, which was exacerbated with cabergoline but improved with bromocriptine.

Cluster‐like headaches have been reported in association with pituitary tumors in at least 10 cases, mostly with prolactinomas 2, 3, 4, 5, 6, 7, 8. Most of them presented as chronic cluster headaches. Nine of them were macroadenomas. Favier et al. 7 reported four cases, but no size of the tumor was available. Three of them are clearly macroadenomas on the MRI. The remaining is reported as intrasellar with spread to the sphenoid sinus and the cavernous sinus on the right side. However, it is not possible to ensure that it is a macroadenoma by the MRI.

The pathogenesis of cluster‐like headaches in association with pituitary tumors is not yet well known. Cavernous sinus invasion with mechanical effect on trigeminal, sympathetic, and parasympathetic nerve fibers may be involved in its pathogenesis. As with our case, in some of the reported cases 2, 6, 8, 9, but not in all 5, cavernous sinus extension of the tumor was concordant with the side of pain attacks. Furthermore, Kallestrup et al. 10 reported a significant association between lateralization of tumor and headaches in 11 patients with pituitary tumors. In contrast, in a study of 63 patients with pituitary tumors, no association between cavernous sinus invasion or tumor volume and headache was observed 11.

In addition, the dopamine–prolactin axis may play a role. This has been suggested by improvement of cluster‐like headaches with dopamine agonists in most cases of prolactinomas 2, 5, 8. Moreover, Negoro et al. 5, Levy et al. 6, and Andereggen et al. 8 reported patients presenting with cluster‐like headaches, which resolved immediately after starting cabergoline, indicating a strong possible relationship between levels of prolactin and cluster‐like headaches. On the other hand, in two cases cabergoline did not improve this type of headache 3, 4. In one of these cases, cabergoline normalized prolactin but did not have any effect on headaches, suggesting that prolactin abnormal levels played no role in its pathogenesis 3.

Mechanism by which prolactin may cause headaches is unknown. It has been reported that headache prevalence is higher in patients with hormone‐secreting pituitary adenomas (prolactin and growth hormone) compared to nonfunctioning pituitary adenomas 11. The pathogenesis of headaches associated with pituitary tumors is complex, and many interactions between hormones and transmitters are proposed. Some authors suggest hypersensitivity of dopamine receptors 12, 13. Furthermore, some authors suggest serotonin hyperfunction as a contributor to dopaminergic dysfunction 14.

It has been reported that prolactin is increased locally in some pain conditions 15, 16, 17. Increased prolactin levels could contribute to the development of certain pain disorders, as it can act as a modulator of sensory trigeminal neurons 18. It can trigger protein kinase C and phosphatidylinositol 3′‐kinase pathways 19. These pathways have been associated with hyperalgesia through activation of capsaicin‐sensitive transient potential receptor vanilliod type 1 (TRPV1) expressed on nociceptors 20, 21, 22. In addition, prolactin has been reported to act on other pain receptors such as transient receptor potential channel ankyrin type 1 (TRPA1) and transient receptor potential channel melastatin type 8 (TRPM8) 16. Further research is warranted to elucidate headache mechanism in pituitary tumors.

The trigeminal autonomic cephalalgia short‐lasting unilateral neuralgiform headache with conjunctival injection and tearing (SUNCT) syndrome has been reported to be induced by dopamine agonists in prolactinomas. Both types of dopamine agonists, bromocriptine and cabergoline, have been implicated in exacerbating trigeminal cephalagias 23, 24. Conversely, one case of SUNCT improved with dopamine agonists 25.

The mechanism by which dopamine agonist produces trigeminal autonomic cephalagias is not known; still, dopamine agonists share some properties with ergot alkaloids that have been shown to alter the trigeminal system 26, 27. In contrast, in our case, cabergoline exacerbated headaches and bromocriptine improved them. The pathogenic mechanism of this effect is unknown. The close temporal relationship between different dopamine agonist's treatment and headache status (exacerbation/improvement) suggests other mechanism rather than effects on dopamine–prolactin axis.

There is evidence implicating serotonin (5‐HT) and its receptors in the pathophysiology of headaches. There is some evidence suggesting impairment of the serotoninergic function in patients with cluster headaches, as well as the role of serotonin on pain regulation mediated through 5‐HT1 and 5‐HT2 receptor subtypes 28, 29; 5‐HT2B/2C receptor antagonists such as propranolol and methysergide are effective for migraine prevention, probably through inhibition of certain pain transmission pathways. Also, methysergide has been recommended for episodic cluster headache; in open studies, it has been shown to be effective in 20–73% of cluster headaches cases 30; 5‐HT1B/D receptor agonists such as triptans have been shown to be effective in the treatment of cluster headaches 31, 32, 33. On the other hand, meta‐chlorophenylpiperazine, a 5‐HT2B/C agonists, has been shown to induce migraine in susceptible individuals 34. We can hypothesize that cabergoline acting as a 5‐HT2B agonist may have worsened cluster‐like headaches, while bromocriptine, a 5‐HT2B antagonist, improved it. Targeting of different serotonin receptors by dopamine agonists may explain the opposite effects on headache status 35.

However, we cannot rule out the possibility that pain relief with bromocriptine could be due to tumor shrinkage. However, the immediate resolution of pain when switching to bromocriptine suggests a pharmacological mechanism rather than a structural one. In addition, she received low doses of cabergoline due to gradual dose escalation till 1 mg/week, which she received only for 1 month when she was changed to bromocriptine.

Furthermore, reported time to decreased size of prolactinomas with dopamine agonists varies widely. It has been reported that the decrease in adenoma size can be detected by imaging as early as 6–8 weeks after initiating therapy 36, 37. However, others have a more delayed response in tumor shrinkage and decrease in tumor size could not be apparent for 6 months.

The hook effect can be seen in two‐site monoclonal “sandwich” assays when the antigens outnumber capture and signal antibodies, binding to both of them and preventing their union. It has been reported with prolactinomas, which can lead to significant therapeutic implications since macroprolactinoma first‐line treatment consists of dopamine agonists, opposed to transsphenoidal surgery for nonfunctioning pituitary adenomas 38. The “hook” effect may appear in patients with prolactin levels after dilution as low as 1320 ng/mL, as in this case 39. This effect can be avoided by performing a 1/100 dilution or using a two‐step processing.

In conclusion, cluster‐like headaches may be associated with pituitary tumors; thus, cluster headaches with atypical presentation should alert for close follow‐up, as well as warrant prolactin level measurement. In addition, cluster headaches should prompt a thorough history and neurological examination. If any abnormality is found, neuroimaging should be performed. In contrast to other reports, there was an opposite response to different dopamine agonists, underscoring our poor understanding of the pathophysiology of pituitary associated headaches. The serotonergic system plays an unclear role in the pathogenesis of cluster headaches, and the activation of different receptors subtypes may yield opposite headache response. A better comprehension of the pathogenesis of cluster‐like headaches associated with prolactinomas is needed, as well as the role the serotonergic system and dopamine agonists play in its regulation. In addition, high‐dose “hook” effect should be ruled out in the setting of large pituitary adenomas with moderate increase in prolactin levels.

## Conflict of Interest

The authors declare that they have no conflict of interest.

## Authorship

MMP: wrote the first draft of the manuscript. GS, MRF, and NS: contributed to the writing of the manuscript. MMP, GS, MRF, and NS: made contributions to the acquisition of the clinical data. MMP, GS, MRF, NS, RP, and CB: agreed with manuscript results and conclusions. MMP, GS, MRF, NS, and CB: jointly developed structure and arguments of the paper. MMP, RP, and CB: made critical revisions and approved final version. All authors: reviewed and approved the final manuscript.
